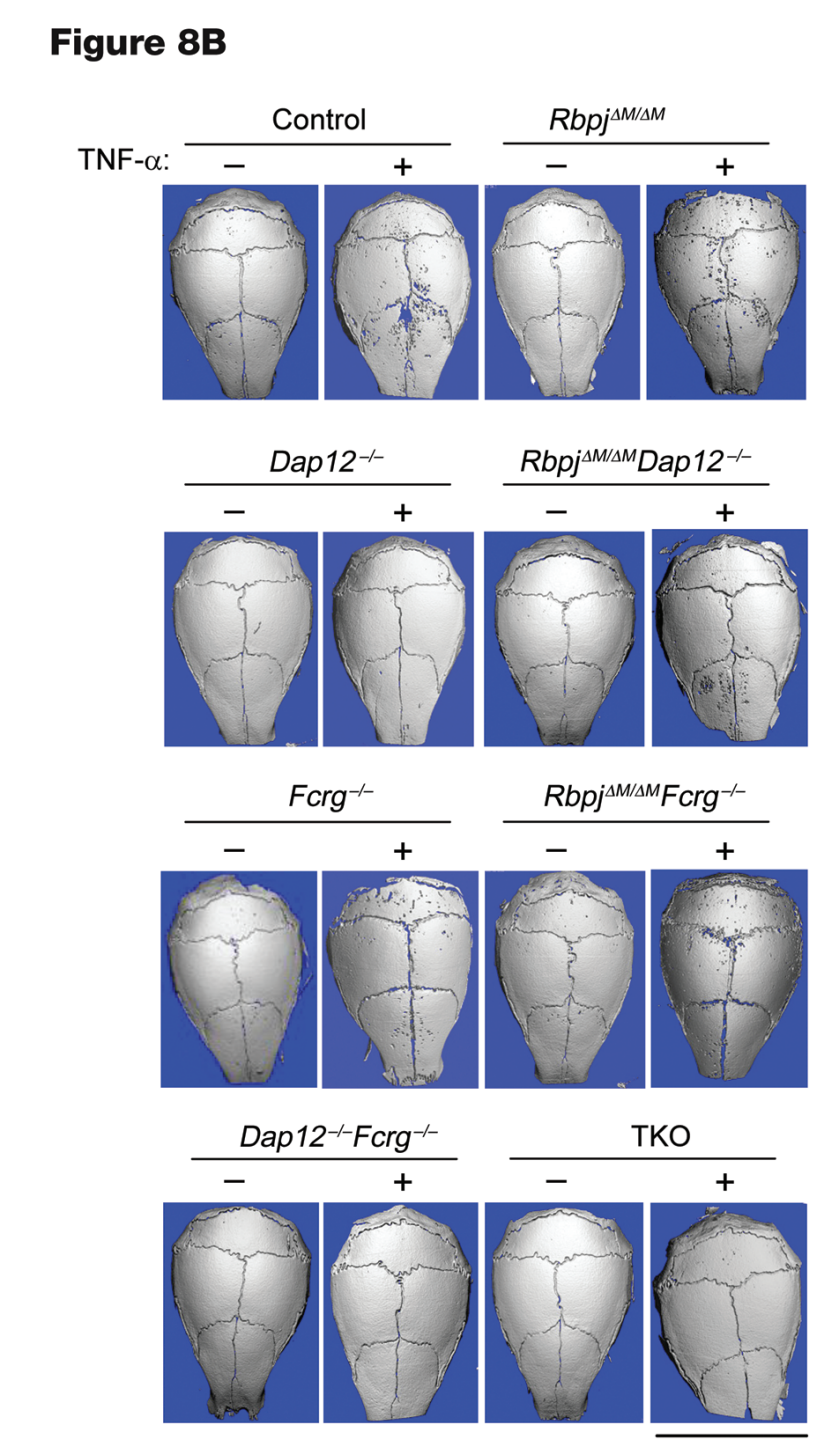# RBP-J imposes a requirement for ITAM-mediated costimulation of osteoclastogenesis

**DOI:** 10.1172/JCI161196

**Published:** 2022-05-16

**Authors:** Susan Li, Christine H. Miller, Eugenia Giannopoulou, Xiaoyu Hu, Lionel B. Ivashkiv, Baohong Zhao

Original citation: *J Clin Invest*. 2014;124(11):5057–5073. https://doi.org/10.1172/JCI71882

Citation for this corrigendum: *J Clin Invest*. 2022;132(10):e161196. https://doi.org/10.1172/JCI161196

The authors recently became aware of errors in Figures 2A, 6G, and 8B. The image presented in the original Figure 2A as the –RANKL, TKO sample was identical to the –RANKL, *Dap12^–/–^ Fcrg^–/–^* sample image in Figure 2A. In addition, the upper and lower p38α blots presented in the right panel of Figure 6G were different exposures of the sample blot. Lastly, in Figure 8B, the same sample was shown for the –TNF condition for the control and *Fcrg^–/–^* panels. The authors have reviewed the original source data and determined that the incorrect–RANKL, TKO sample was shown in Figure 2A, the incorrect p38α blot was presented in the lower right panel of Figure 6G, and the incorrect –TNF, *Fcrg^–/–^* image was shown in Figure 8B. The corrected versions are shown below. The authors also wish to disclose that in Figure 6A, the same β-tubulin loading control (same samples) is shown for the top left and bottom left panels and serves as a control for the p-PLCγ2, NFATc1, and c-FOS panels. The authors have stated that the corrections do not affect the conclusions of the article.

The authors regret the errors.

## Figures and Tables

**Figure:**
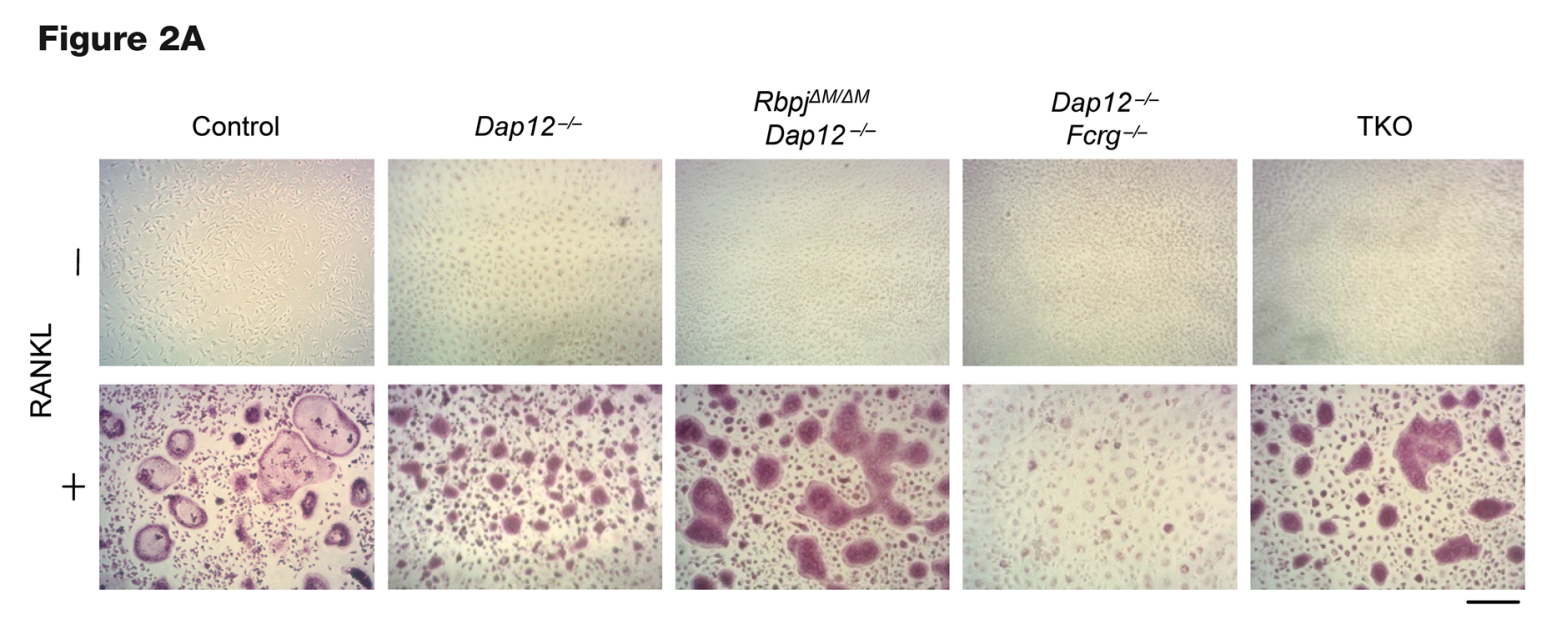


**Figure:**
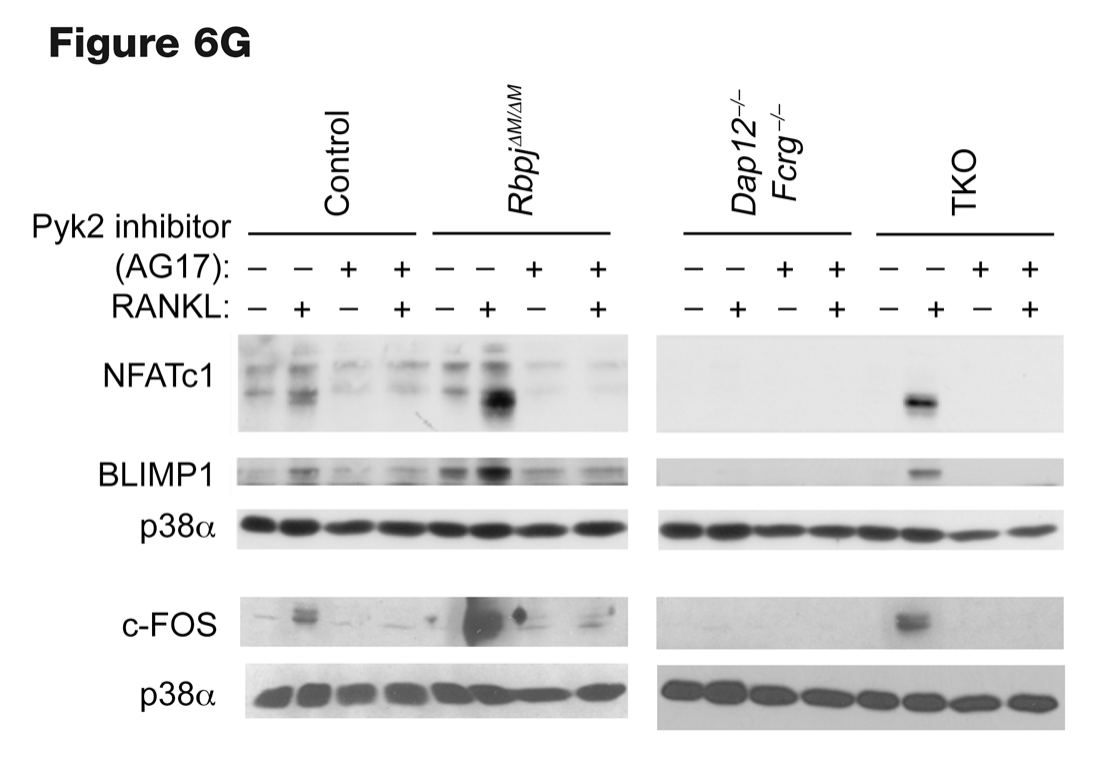


**Figure:**